# *COLEC10* is mutated in 3MC patients and regulates early craniofacial development

**DOI:** 10.1371/journal.pgen.1006679

**Published:** 2017-03-16

**Authors:** Mustafa M. Munye, Anna Diaz-Font, Louise Ocaka, Maiken L. Henriksen, Melissa Lees, Angela Brady, Dagan Jenkins, Jenny Morton, Soren W. Hansen, Chiara Bacchelli, Philip L. Beales, Victor Hernandez-Hernandez

**Affiliations:** 1 Genetics and Genomic Medicine Programme, UCL Great Ormond Street Institute of Child Health, London, United Kingdom; 2 Department of Cancer and Inflammation Research, Institute of Molecular Medicine, University of Southern Denmark, Odense, Denmark; 3 Department of Clinical Genetics, Great Ormond Street Hospital, London, United Kingdom; 4 North West Thames Regional Genetics Service, Kennedy-Galton Centre, Northwick Park Hospital, London, United Kingdom; 5 Department of Clinical Genetics, Birmingham Women’s Hospital, Birmingham, United Kingdom; University of Oxford, UNITED KINGDOM

## Abstract

3MC syndrome is an autosomal recessive heterogeneous disorder with features linked to developmental abnormalities. The main features include facial dysmorphism, craniosynostosis and cleft lip/palate; skeletal structures derived from cranial neural crest cells (cNCC). We previously reported that lectin complement pathway genes *COLEC11* and *MASP1/3* are mutated in 3MC syndrome patients. Here we define a new gene, *COLEC10*, also mutated in 3MC families and present novel mutations in *COLEC11* and *MASP1/3* genes in a further five families. The protein products of *COLEC11* and *COLEC10*, CL-K1 and CL-L1 respectively, form heteromeric complexes. We show *COLEC10* is expressed in the base membrane of the palate during murine embryo development. We demonstrate how mutations in *COLEC10* (c.25C>T; p.Arg9Ter, c.226delA; p.Gly77Glufs*66 and c.528C>G p.Cys176Trp) impair the expression and/or secretion of CL-L1 highlighting their pathogenicity. Together, these findings provide further evidence linking the lectin complement pathway and complement factors *COLEC11* and *COLEC10* to morphogenesis of craniofacial structures and 3MC etiology.

## Author summary

The 3MC syndrome is a unifying term amalgamating four rare recessive genetic disorders with overlapping features namely; Mingarelli, Malpuech, Michels and Carnevale syndromes. It is characterised by facial malformations including, high-arched eyebrows, cleft lip/palate, hypertelorism, developmental delay and hearing loss. We previously reported that lectin complement pathway genes *COLEC11* and *MASP1/3* were mutated in 3MC syndrome patients. Here we describe a new gene from the same pathway, *COLEC10*, mutated in 3MC patients. Our results show that *COLEC10* is expressed in craniofacial tissues during development. We demonstrate how CL-L1, the protein expressed by *COLEC10*, can act as a cellular chemoattractant *in vitro*, controlling cell movement and migration. We overexpressed constructs carrying *COLEC10* non-sense mutations found in our patients, CL-L1 failed to be expressed and secreted. Moreover, when we expressed a missense *COLEC10* construct, CL-L1 was expressed but failed to be secreted. In sum, we discovered a new gene, *COLEC10*, mutated in 3MC syndrome and we propose a pathogenic mechanism for 3MC relating to the failure of CL-L1 function and its craniofacial developmental consequences.

## Introduction

3MC syndrome (MIM 257920;265050;248340) is a unifying term amalgamating four rare autosomal recessive disorders with overlapping features namely; Mingarelli, Malpuech, Michels and Carnevale syndromes. 3MC syndrome is characterized by facial features including hypertelorism, cleft lip/palate, high-arched eyebrows, craniosynostosis, developmental delay and hearing loss [1–3]. We previously reported that mutations in *COLEC11* and *MASP1/3* genes were responsible for several cases of 3MC syndrome [4]. Since then, further novel mutations in *MASP1/3* and *COLEC11* have been reported in 3MC patients [5–7]. *COLEC11* and *COLEC10* encode CL-K1 (also known as CL-11) and CL-L1 (also known as CL-10) respectively, members of the collectin family with an N-terminal collagen-like domain linked to C-terminal carbohydrate-recognition domains (CRDs). CL-K1 and CL-L1 are able to bind to microorganisms including bacteria, fungi and viruses, through their CRDs. This binding capacity to antigens, followed by their interaction with MASP proteins, is their main role in lectin complement pathway activation. [8–12]. However the pathogenic mechanism of lectin complement related proteins in 3MC syndrome is not yet understood [4].

CL-K1 and CL-L1 can also work in partnership in complement activation [13]. Usually CL-K1 and CL-L1 form homodimers, as is generally the case with CDR-domain containing proteins but CL-K1 and CL-L1 can form CL-K1/CL-L1 (also known as CL-LK) heterodimers in plasma and *in vitro*. These CL-LK heterodimers can also interact and form complexes with MASP-1, MASP-2 and MASP3 [10].

*MASP1/3* encodes for 3 alternative products MASP-1, MASP-3 and MAp44 [14]. MASP-1 collaborates with MASP-2 to activate C4. MAp44 has the MASP1 H domain truncated and inhibits MASP1 and MASP2 complement activation. MASP3 shares H chain domain with MASP1 and have a unique protease domain. The precise role of MASP-3 in complement signalling is still unclear, but it has been proposed to form a complex with CL-LK and MASP-2 [10]. It remains to be determined whether these interactions play a role in embryological development, perturbation of which gives rise to the diverse morphological features of 3MC syndrome.

Recently it has been shown that 3MC mutations in *COLEC11* inhibit secretion of CL-K1 in mammalian cells, reducing the normal serum levels of CL-K1 and probably disrupting interaction with MASPs or CL-L1 [15].Another report describes how three exonic polymorphisms in *COLEC11* and *COLEC10* also have an effect in reducing levels of circulating CL-K1 and CL-L1 in serum [16]. Those findings hint how mutations and polymorphisms in both *COLEC11* and *COLEC10*, can directly affect CL-K1 and CL-L1 secretion.

The skeletal phenotype of 3MC patients is the result of complex embryological processes, including neural crest cell (NCC) induction, migration, morphogenesis and differentiation [17]. Correct migration of cNCC is essential for the formation of many tissues in the head from cartilage and bones to muscle and ganglia [18–21]. The regulation and control of NCC migration is complex involving multiple genetic pathways including Wnt, Shh and transcription factors such as *Hox* and *Dlx* genes [18,22,23]. Complement factors, such as C3a, have been recently established to play a role in NCC cohesion during migration. Mayor and collaborators have established how complex collective cell migration of NCC requires complement proteins. For example, C3a and its receptor C3aR work together to co-attract each other in order to maintain the coordinated migration of NCC [24–26].

In the present study we describe mutations in a novel lectin alternative pathway gene, *COLEC10*, in 3MC patients, adding to the body of evidence implicating the complement pathway in human development. We also present new *COLEC11* and *MASP1/3* mutations found in our cohort of 3MC patients. To validate *COLEC10* mutations as causative of 3MC syndrome we determine its expression pattern in the developing mouse embryo and we further demonstrate the *in vitro* functional consequences of *COLEC10* mutations, and present evidence that CL-L1 act as a cellular chemoattractant. Finally we propose a pathogenic mechanism for 3MC relating to the failure of CL-L1 function and its developmental consequences in 3MC.

## Results

### Exome sequencing reveals mutations in COLEC10 as a cause of 3MC

We collected a bank of patient DNA samples comprising diagnoses of Carnevale, Mingarelli, Michels and Malpuech syndromes. Our cohort currently consists of 45 3MC families of Asian, Middle Eastern and European origin. We previously demonstrated that mutations in *COLEC11* and *MASP1/3* lectin complement pathway related genes are causative of 3MC syndrome in 11 families and 16 patients. Therefore, we screened for *COLEC11* and *MASP1/3* mutations by Sanger sequencing in the remaining 34 families and 36 patients in this heterogenous group of patients.

**Fig 1:**
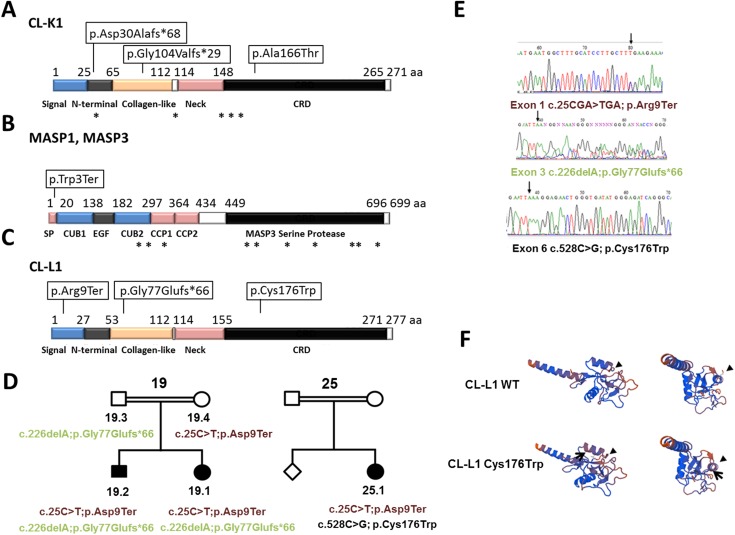
New 3MC mutations in *COLEC11*, *MASP1/3* and *COLEC10* A-C. Summary of 3MC mutations position in CL-K1, MASP1, MASP3 and CL-L1 proteins. Boxes indicate position of new mutations described in this work. Asterisks indicate already known mutations. D. Pedigrees of families 19 and 25 with *COLEC10* mutations. *COLEC10* mutations are indicated under carriers and affected patients. E. Chromatogram of new *COLEC10* mutations. F. Tridimensional structure by SWISS-MODEL Workspace for Wild-type and Cys176Trp mutated CL-L1. On the left, lateral view of the second helix-loop-helix domain. This domain is part of the C-lectin type domain. On the right, forward view. Arrow indicates the position of the missense change p.Cys176Trp. The structure of CL-L1 c-lectin domain changes in p.Cys176Trp mutants (arrowheads).

We found three novel homozygous mutations in *COLEC11* (NM_024027.4) in three patients and a single homozygous mutation in *MASP1/3* (NM_139125.3) in one patient (see Table 1 and Fig 1A and 1B). Of these, two patients were from consanguineous families; MC35.1 (Pakistani) and MC37.1 (Somalian). Both harbored non-synonymous homozygous mutations in *COLEC11* leading to a predicted premature termination codon, c.309delT (p.Gly104Valfs*29, exon 4) and a predicted damaging missense, c.G496A (p.Ala166Thr, exon 6) respectively. For patient M35.1 we sequenced the parents, demonstrating that the mutations segregated with the disorder. Parental samples were not available for patient MC37.1.

**Table 1:** New *COLEC11* and *MASP1/3* mutations in 3MC patient cohort

**Family**	**Subject**	**Diagnosis**	**Origin**	**Mutated gene**	**Nucleotide change**	**Protein change**	**Control chromosomes allele frequency**
**MC29**	29.1	Carnevale	United Arab Emirates	*COLEC11*	HMZ 89_98delATGACGCCTG	p.Asp30Ala*fs68	Not in ExAc
**MC35**	35.1	Carnevale	Pakistan	*COLEC11*	HMZ c.309delT	p.Gly104Valfs*29	Not in ExAc
**MC37**	37.1	Carnevale	Somalia	*COLEC11*	HMZ c.G496A	p.Ala166Thr (Polyphen damaging)	Not in ExAc
**MC27**	27.1	Carnevale	Pakistan	*MASP1/3*	HMZ c.9G>A	p. Trp3Ter	0.000008389

We found in patient MC29.1 a deletion of 10 nucleotides in *COLEC11* (c.89_98delATGACGCCTG, exon 2) which predicts a frameshift change and the introduction of a premature stop codon (p.Asp30Alafs*68). None of the *COLEC11* mutations was present in the Exome Aggregation Consortium Database (ExAC), (Cambridge, MA URL http://exac.broadinstitute.org). Overall, two of the new *COLEC11* mutations lead to premature terminations (p.Gly104Valfs*29 and p.Asp30Alafs*68), or the missense mutation p.Ala166Thr. This last missense change lies, within the CRD, as shown in Fig 1A, and probably disrupts its recognition function.

In our 3MC cohort we also found a new mutation affecting the second previously described gene mutated in 3MC, *MASP1/3* (NM_139125.3). Patient MC27.1, with a consaguinous family, presents a homozygous nonsense mutation (c.9G>A) leading to premature truncation of the protein recently been reported by [6].

These results corroborate our previous finding that genes involved in the lectin complement pathways cause 3MC. However, mutations in *COLEC11* and *MASP1/3* were excluded in the remaining 30 families and 32 patients. Therefore, we performed whole exome sequencing (WES) in six 3MC patients from consanguineous families, without mutations in *COLEC11* or *MASP1/3*, in order to identify new causative gene associations. We found one patient diagnosed with Michels syndrome harbouring deletions in *COLEC10* (NM_006438.4), another member of the collectin family. Despite parental consanguinity in this family, we discovered that the proband, MC19.1 harboured compound heterozygous mutations, c.25C>T; p.Arg9Ter in exon 1 and c.226delA; p.Gly77Glufs*66 in exon 3. We confirmed these mutations segregated with disease by Sanger sequencing (Table 2 and Fig 1D and 1E). The affected sibling, MC19.2, also harboured the same compound heterozygous mutations in *COLEC10*.

**Table 2:** New *COLEC10* mutations found in families MC19 and MC25

**Family**	**Subject**	**Diagnosis**	**Origin**	**Mutated gene**	**Nucleotide change**	**Protein change**	**Control chromosomes allele frequency**
**MC19**	19.1	Michels	Pakistan	*COLEC10*	c.25C>T; c.226delA	p. Arg9Ter; p.Gly77Glufs*66	0.00003300 Not in ExAc
**MC19**	19.2	Michels	Pakistan	*COLEC10*	c.25C>T; c.226delA	p. Arg9Ter p.Gly77Glufs*66	0.00003300 Not in ExAc
**MC25**	25.1	Malpuech	Pakistan	*COLEC10*	c.25C>T c.528C>G	p. Arg9Ter p.Cys176Trp	0.00003300 0.000008273

Next we Sanger sequenced *COLEC10* in the remainder of our patient cohort. These patients were previously screened for *COLEC11* and *MASP1/3* mutations, with none identified. We identified another patient (25.1) with the p.Arg9Ter *COLEC10* mutation accompanied by a new missense mutation c.528C>G, p.Cys176Trp (exon6) in the other allele (Table 2 and Fig 1D and 1E). The unaffected sibling or parents were not available for testing, therefore we cannot conclusively state that both mutations in patient 25.1 could be in -cis.

The p.Gly77Glufs*66 mutation is not present in the ExAC database and p.Cys176Trp (position Chr8:120118124 C / G, not found in dbSNP) has a frequency of 1 in 120850 chromosomes in the same database. The p.Arg9Ter mutation (rs149010496) is present in only 4 alleles out of 121220 (ExAC). Collectively, these data strongly support the notion that pathogenic mutations in *COLEC10* cause a subset of 3MC diagnoses.

*COLEC10* mutations c.25C>T; p.Arg9Ter and c.226delA; p.Gly77Glufs*66 both lead to early termination and are likely to produce either truncated proteins or undergo non-sense mediated decay. However, the missense mutation p.Cys176Trp lies in the CRD domain of CL-L1 (Fig 1C), affecting a cysteine residue Cys176 that forms a disulphide bond with C270 [9] and is predicted by PolyPhen-2 to be damaging (http://genetics.bwh.harvard.edu). We next used the SWISS-MODEL Workspace application (http://swissmodel.expasy.org) to predict how the p.Cys176Trp mutation might affect the secondary structure of the CL-L1 protein. Residue 176 on the second helix-loop-helix domain of the protein is predicted to change the tridimensional structure of the protein (Fig 1F), probably affecting the C-type lectin domain function. Table 3 shows detailed clinical features for all of described patients.

**Table 3:** Detailed clinical features for all 3MC described patients.

3MC Clinical Features	MC19.1	MC19.2	MC25.1	MC27.1	MC29.1	MC35.1	MC37.1
***Demographics***							
Sex	F	M		M	M		
Age (yrs at time of study)	21	17					
Country of origin	Pakistan	Pakistan	Pakistan	Pakistan	Lebanon		Somalia
Consanguinity	Y	Y	Y	Y	Y		Y
***Stature***							
Small (<3rd centile)	Y	Y	N	N	N	N	N
***Craniofacial features***							
Arched eyebrows	N	N	N	Y	Y	Y	Y
Blepharoptosis	Y	Y	Y	Y	Y	Y	N
Epicanthus inversus	Y	Y	Y	Y	N	Y	N
Hypertelorism	N	N	N	Y	N	Y	N
Dysplastic ears	N	N	Y	N	N	N	Y
Ear pit(s)	N	N	Y	N	N	N	N
Cleft lip (unilateral)	N	Y	N	N	N	N	N
Cleft lip (bilateral)	N	N	Y	Y	N	N	N
Cleft palate (unilateral)	N	Y	N	N	N	N	N
Cleft palate (bilateral)	N	N	Y	Y	N	N	N
***Development***							
Developmental delay	N	N	N	Y	N	N	N
Hypotonia	N	N	N	N	N	N	Y
***Trunk and limbs***							
Radio-ulnar synostosis	N	N	N	N	Y	N	Y
Pre-axial polydactyly	N	Y	N	N	N	N	N
Diastasis recti/Umbilical hernia	N	N	N	N	Y	Y	Y
Sacral dimple/crease	N	N	Y	Y	N	Y	N
Clinodactyly	N	N	Y	N	N	N	N
***Cardiovascular system***							
VSD	N	N	N	N	N	N	N
ASD	N	N	N	N	N	N	N
PDA	N	N	N	Y	N	N	N
***Renal anomalies***							
Horseshoe kidney	N	N	N	N	N	N	Y
Other							
Micropenis	N	N	N	Y	N	N	N
Undescended testes	N	N	N	Y	N	N	N
Corneal clouding	N	N	N	N	N	N	Y
Deep set nails	N	N	N	N	N	N	Y
Feeding difficulties	N	N	N	N	N	N	Y

Y = Feature is present. N = Feature is not present.

### Expression of COLEC10 in cells and mouse embryos

**Fig 2:**
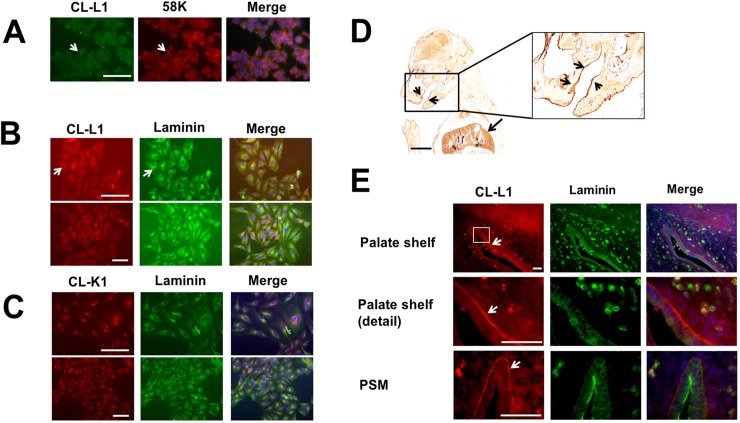
Cellular and embryonic localisation of CL-L1. A. Immunostaining of ATDC5 cells with the golgi marker 58K and CL-L1. CL-L1 shows localisation with golgi apparatus (white arrow). B. Laminin and CL-L1 coimmunolocalisation. Laminin shows partial cellular immunolocalisiton with CL-L1 around the golgi area (arrows). Scale bar 50 μm C. Laminin and CL-K1 coimmunolocalisation. CL-K1 staining shows a very strong golgi localisation with partial cytoplasmatic laminin colocalisation. D. CL-L1 immunohistochemistry of a 18.5 days postfertilisation mouse embryo. CL-L1 is expressed in the liver (long arrow) and submucosal patal region (short arrows). E. Immunofluorescence showing co-localisation of CL-L1 and Laminin in E13.5 mouse embryos sections. CL-L1 is expressed in the basal membrane of the ephithelium in the palate shelf of the maxilla (arrows). In contrast Laminin expression is present all around the ephitelium membrane. A faint but clear CL-L1 expression is also observed in the cytoplasm of the epithelium and in the mesenchyme of the palate. PSM; rostral extremity of right palatal shelf of maxilla. Scale bar 100 μm.

To further characterise the function of *COLEC10* we assessed intracellular localisation of CL-L1 in ATDC5 cells, a murine chondrocyte cell line. Consistent with previous results for *COLEC11* [4], we observed expression of CL-L1 in the Golgi apparatus consistent with a secreted peptide, colocalising with the TGN marker 58K, and with cytosolic expression (Fig 2A). We also found CL-L1 colocalised with laminin, a major component of the basal lamina (Fig 2B). This expression is similar to the cellular colocalisation we found between CL-K1 and laminin (Fig 2C).

Next, we analysed the expression of CL-L1 during murine craniofacial development. We detected CL-L1 expression in the epithelium and mesenchyme of the palate shelf and jaw in E18.5 embryos (Fig 2D). Moreover, we found by immunofluorescence that this particular mandibular epithelial expression is present as early as E13.5, revealing coexpression between CL-L1 and laminin, where CL-L1 is clearly visible in the basement membrane in the palate area (Fig 2E).

### CL-L1 regulates development of craniofacial structures acting as a migratory chemoattractant

**Fig 3:**
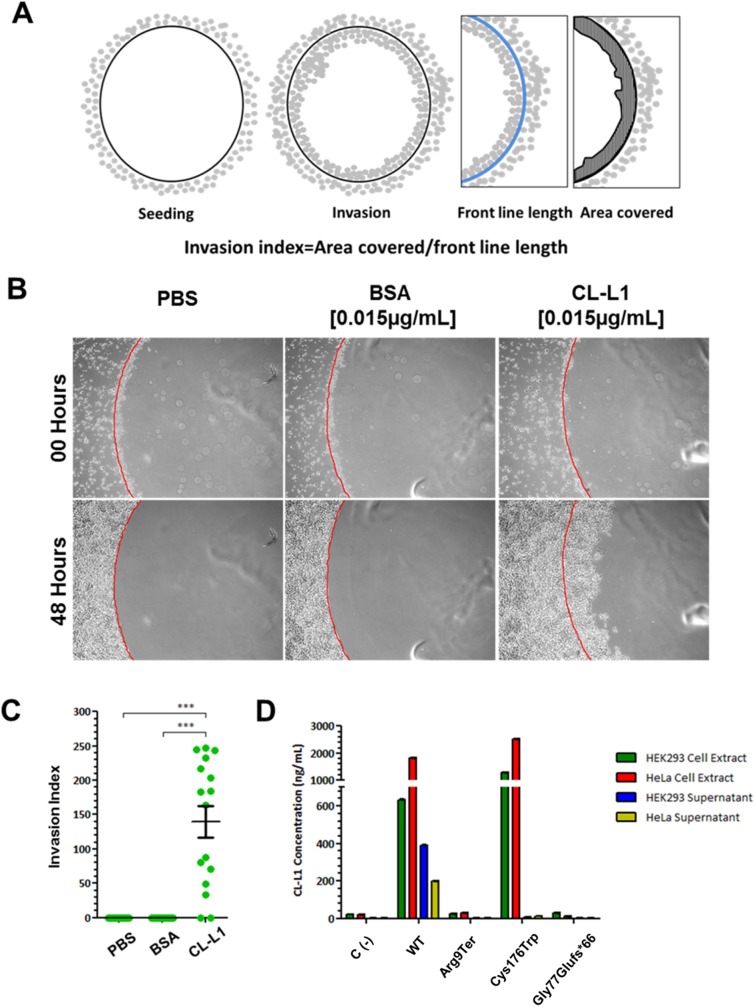
CL-L1 regulates cell migration and act as migratory chemoattractant. A. Experimental design for cell migration assay and invasion rate. Agarose spots containing CL-L1 and/or CL-K1 were attached to the glass and HeLa cells seeded. After 24 hours pictures were taking on different spots. The area covered inside the spot by the cells (advance front) was measured and normalised for the length of the perimeter of the spot. B. Representative pictures of agarose spots containing PBS, BSA and CL-L1 with cells invading 48 hours after seeding after. Note how HeLa cells are attracted and invade the agarose sport containing CL-L1. C. Quantification of the invasion ratio. HeLa cells when exposed to CL-L1 cells were more attracted to invade the agarose spots than PBS controls. D. ELISA results in HeLa and HEK293 cell pellets and supernatants after *COLEC10* mutant construct transfections. *COLEC10* Wild-type (*COLEC10*^WT^) and three *COLEC10* mutations (*COLEC10*^*ArgXTer*^, *COLEC10*^Gly77Glufs*66^ and *COLEC10*^Cys176Trp^) cDNAs where cloned and transfected in HEK293 and HeLa cells. Untransfected cells C(-) were used as a control to prove no endogenous CL-L1 was affecting the readings. Concentration of expressed CL-L1 was tested by ELISA in cells extracts and cell supernatant. CL-L1 was found in *COLEC10*^WT^ and *COLEC10*^Cys176Trp^ cell extracts in both cell types, but no CL-L1 was expressed after transfecting *COLEC10*^*ArgXTer*^ or *COLEC10*^Gly77Glufs*66^ constructs or in the untransfected C(-) cells. *COLEC10*^WT^ and *COLEC10*^Cys176Trp^ showed very similar levels of CL-L1 expression in HeLa and HEK293 cell types, with slightly higher expression in *COLEC10*^Cys176Trp^, (2518 ng/ml HeLa and 1302 ng/ml HEK293) versus *COLEC10*^WT^ (1823 ng/ml HeLa and 632 ng/ml HEK293). However, the levels of CL-L1 were undetectable in the supernatant of *COLEC10*^Cys176Trp^ transfections, in contrast with *COLEC10*^WT^ transfected cells (200 ng/ml HeLa and 390 ng/ml HEK293)

We investigated the ability of CL-L1 to act as a chemoattractant in the context of human cells. We spotted 1% (w/v) low melting point agarose discs mixed with PBS, BSA or recombinant human CL-L1. As reported previously, when the same experiment was performed for CL-K1 [4], cells were observed to invade the protein-containing agarose disc. To quantify this effect, we calculated the cell invasion index as shown in Fig 3A. We found that PBS and BSA containing discs failed to attract any cells (Fig 3B and 3C and S1 and S2 Movies respectively) which was in stark contrast to CL-L1 containing discs that exhibited extensive migration/invasion into the discs with an invasion index score of 140.0±22.9 (Fig 3C and S3 Movie).

### COLEC10 mutations inhibit secretion of CL-L1

Having demonstrated a role for CL-L1 in normal craniofacial development we sought to confirm that the mutations found in our 3MC patients were pathogenic. We predicted that *COLEC10* mutations c.25C>T; p.Arg9Ter and c.226delA; p.Gly77Glufs*66 would lead to either truncated or absent protein. However, we expected that the missense mutation c.528C>G, p.Cys176Trp, affecting a crucial cysteine residue, would likely lead to abnormal protein folding and possibly affects secretion, as seen with three disease-associated mutations in *COLEC11* [15]. To test this hypothesis, we transfected *COLEC10*^WT^, *COLEC10*^Arg9Ter^ and *COLEC10*^Gly77Glufs*66^ constructs into HeLa and HEK293 cell lines and detected CL-L1 expression.

Immunoblotting demonstrated that CL-L1 protein was present in both cell extracts and supernatants when *COLEC10*^WT^ plasmid was transfected into HEK293 cells. By contrast, no protein was detected when the mutant plasmids *COLEC10*^Arg9Ter^ and *COLEC10*^Gly77Glufs*66^ were transfected, suggesting that both transcripts underwent nonsense-mediated decay. Transfection of *COLEC10*^*Cys176Trp*^ plasmid allowed CL-L1 expression but not secretion as demonstrated by detection of CL-L1 in the cell lysates but not in the supernatant (S1 Fig).

Western blot data were further supported by quantitative ELISA (Fig 3D). The results showed highest levels of CL-L1 protein in pellets of cells transfected with *COLEC10*^*Cys176Trp*^ plasmid than cells transfected with *COLEC10*^WT^ (HeLa *COLEC10*^*Cys176Trp*^ 2518.3±21.3ng/mL vs HeLa *COLEC10*^WT^ 1823.3±7.2ng/mL, p<0.001; HEK293 *COLEC10*^*Cys176Trp*^ 1302.7±3.7ng/mL vs HEK293 *COLEC10*^WT^ 632.0±3.6ng/mL, p<0.001. Fig 3D and S1 Table). However, secretion of CL-L1 was severely reduced in the *COLEC10*^*Cys176Trp*^ transfected cells compared with *COLEC10*^WT^ supernatant transfections (HeLa *COLEC10*^*Cys176Trp*^ 12.5±0.2ng/mL vs HeLa *COLEC10*^WT^ 200.3±1.5ng/mL, p<0.001; HEK293 *COLEC10*^*Cys176Trp*^ 5.7±0.1ng/mL vs HEK293 *COLEC10*^WT^ 390.2±4.1ng/mL, p<0.001. Fig 3D and S1 Table). These results suggest that accumulation of CL-L1 in cell pellets in *COLEC10*^*Cys176Trp*^ is the result of severely reduced levels of CL-L1 secretion. Besides, no CL-L1 expression was observed for *COLEC10*^Arg9Ter^ and *COLEC10*^Gly77Glufs*66^ transfected cells, which served as a negative control.

## Discussion

We previously showed *COLEC11* and *MASP1/3* lectin alternative pathway genes were mutated in 3MC patients. Since our initial discovery, several groups reported mutations in *COLEC11* and *MASP1/3* in their 3MC cohorts [5–7]. Here we report four new mutations for *COLEC11* affecting four further 3MC patients from consanguineous families. None of these mutations has been found in the ExAc database, supporting pathogenicity and indicating their private nature in these pedigrees. We also identified another *MASP1/3* mutation in the homozygous state, c.9G>A, in our cohort confirming a prior report of this mutation by Urquhart *et al*. [6].

These results increase the percentage of patients with known mutations in our 3MC cohort; 23% carry a *COLEC11* mutation and 12% now carry a *MASP1/3* mutation. In the remaining patients we identified a second member of the collectin family, *COLEC10*, found to be mutated in 3MC. The addition of these 2 families in *COLEC10* (5%) increase the coverage to 40% of known genes of our patients. Therefore, over 60% of our 3MC cohort is still without molecular confirmation of disease and that at least one further gene remains to be identified.

In contrast with *COLEC11* patient mutations, all three *COLEC10* patients have compound heterozygous *COLEC10* mutations, which is slightly surprising as they come from consanguineous families. They all share the terminating mutation c.25C>T;Arg9Ter, found in ExAc in the general population at a low frequency (0.00003300) (Table 2), whereas the mutations c.226delA and c.528C>G were not present in the ExAc database.

In recent years a very well documented evidence implicating cNCC migration in craniofacial cartilage and bone morphogenesis has accumulated (reviewed in [19]). Our data suggests the failure of NCCs to migrate correctly is the principal factor leading to craniofacial abnormalities in 3MC patients. We confirmed that CL-L1 has chemotactic properties, most likely through recognition of carbohydrates on the cell surface, providing a potential explanation on how its absence can lead to abnormal NCC migration in 3MC. This is not surprising as other complement pathway proteins have previously been shown to play important roles in cell migration. For example in the first steps of the regulation of NCCs, crest cells are co-attracted by the complement fragment C3a and its receptor C3aR. When the C3aR function is inhibited enteric neural crest cell adhesion and migration is affected, and there is an increase in NCC dispersion [24,26]. It is worth noting that the lectin complement pathway can also induce cleavage of C3 to C3a [25] which in turn can regulate NCC migration.

Furthermore, other complement factors also regulate cell migration and morphology. C3 regulates epithelial-mesenchymal transition via *TWIST1* activation [27]. C3a also controls radial intercalation during early gastrulation and tissue spreading [28]. An important common functionality of C3a is its capacity to act as a chemoattractant to pull cells together and force them to migrate collectively.

In the lectin complement pathway CL-L1 can form a complex with CL-K1, called CL-LK, and bind to MASP1/3 and MASP2 [10] to activate the lectin complement pathway.

We propose here that the role of CL-L1 and CL-K1 lies in regulating cell migration via cell attraction in 3MC syndrome. We know that CL-L1 and CL-K1 can act by themselves to attract cells but both can also form the heteromeric complex CL-LK that can also bind to MASP1/3 and MASP2 with higher affinity than CL-K1 homodimers [10]. Therefore, it is possible that the NCC migration *in vivo* requires cooperation of heteromeric interactions between CL-L1 and CL-K1. That is supported by the observations that *COLEC11* and *COLEC10* genetic variants strongly influence the circulating serum levels of CL-K1 and CL-L1 and that a major proportion of these proteins are circulating in the form of heterocomplexes [16]. As such, whilst we have demonstrated CL-L1 can in itself induce cell migration and invasion, the exact molecular pathway leading to NCC migration regulation requires further investigation.

We did not observe any *COLEC10* expression in cells pellets and supernatant when overexpressing the mutations 9G>A; ArgXTer and c.226delA; p.Gly77Glufs*66 (Fig 3D). However, the missense c.528C>G, p.Cys176Trp mutation did not affect *COLEC10* expression, although it did prevent cellular secretion of the protein into the supernatant. Furthermore, 3MC patient mutations in *COLEC11* also show a similar secretory phenotype disruption [15]. These data suggest that the mechanism of disease could be linked to abnormal CL-L1 secretion. The fact that we observe continuous expression of CL-L1 in E13.5 embryos and P0 pups in the mandibular epithelium could indicate there is an additional role for maintaining cellular adhesion even after NCC migration is complete; further data are required to prove this hypothesis.

In summary, we have described here a new gene, *COLEC10*, that when mutated causes 3MC syndrome. Further mutations identified in *COLEC11* and *MASP1/3* further confirm clinical suspicions of disease in several 3MC patients but leaves a sizeable proportion (60%) without molecular confirmation and implicate one or more further genes. We propose that the lectin complement pathway acts as a chemottractant to guide and possibly to maintain cNCC adhesion. We believe that in future more genes linked to the lectin complement pathway and with roles in cellular adhesion and guidance will be found to be mutated in 3MC syndrome patients and other craniofacial conditions.

## Materials and methods

### Exome capture

Patients and families samples were screened by whole-exome sequencing, including the proband and both parents when available. In each case, genomic DNA was enriched for exonic regions using the SureSelect All Exon 50Mb Targeted Enrichment kit (targeting 202,124 exons from 20,718 genes) from Agilent Technologies, according to the manufacturer's protocol. Captured libraries were sequenced on an Illumina HiSeq 2000 instrument using Illumina sBot clustering and HiSeq chemistries v1.0, under a paired-end 100-bp read-length protocol, with four samples per flow cell lane to achieve minimum median coverage of 60×. All exomes for *COLEC11*, *COLEC10* and *MASP1/3* have a coverage of at least x15. For specific exonic coverage of 3MC family 19 see S1 Methods Table. The variant annotation and interpretation analyses were generated through the use of Ingenuity Variant Analysis software version 3.1.20140902 from Ingenuity Systems. For the recessive model, homozygous/compound heterozygous variants in the affected individual were retained. Intronic and exonic synonymous variants were filtered out; exonic and splice variants (up to 2 base pairs into intron or predicted pathogenic on MaxEntScan) with a public databases (ExAC, 1000 Genomes and ESP Exomes) frequency <0.01% (3MC phenotype) were retained. All disease causing variants (*COLEC10*) were validated by Sanger sequencing. Filtering pipelines for variants, ingenuity and a final list of all variants identified are presented in S2 Methods Table, S3 Methods Table and S4 Methods Table.

### Cell culture

HEK293 and HeLa cells were cultured in DMEM (Invitrogen) supplemented with 10% (v/v) foetal bovine serum and incubated in humidified 5% CO2 at 37oC.

### Cell migration assay

An agarose spot assay was used to assess chemotactic invasion potential of CL-L1. Briefly, a 2% (w/v) solution of low-melting point agarose (Invitrogen) in phosphate-buffered saline was boiled and when the solution cooled to around 50oC it was mixed 1:1 with solutions of PBS, bovine serum albumin (BSA), recombinant CL-K1 (Abnova, H00078989-P01) and/or recombinant CL-L1 (Abnova, H00010584-P01). 10μL of the agarose-protein mix was then spotted onto the wells of plastic tissue culture plates, allowed to polymerise at room temperature for around 10 minutes and cells added. Cell migration and invasion was monitored at 37°C with 5% CO2 for around 48 hours using an Axiovert 135 microscope (Zeiss) equipped with a motorized stage that captured 1 image per 15 minutes (Volocity software v6.3, PerkinElmer). Migration and invasion was quantified using ImageJ software by measuring the area within the agarose-protein discs that had been occupied by cells (Fig 3A).

### Transfection

Patient mutations c.25C>T,p.Arg9Ter; c.226delA,p.Gly77Glufs*66 and c.528C>G;p.Cys176Trp were introduced into a plasmid encoding wild-type human CL-L1 (pCMV6-XL5-COLEC10; OriGene, SC303774) using QuickChange II Site-Directed Mutagenesis kit (Agilent) with hCOLEC10Arg9Ter, hCOLEC10Gly77Glufs*66 and hCOLEC10Cys176Trp primers (S5 Methods Table). hCOLEC10WT, hCOLEC10Arg9Ter, hCOLEC10Gly77Glufs*66 and hCOLEC10Cys176Trp plasmids were complexed with 25kDa branched polyethylenimine (Sigma) and transfected into HEK293 and HeLa cells. A negative control with untransfected HEK293 and HeLa cells was used to show CL-L1 expression was not innate cell endogenous expression.

### Western blot

Western blot was performed using standard protocols. Briefly, 48 hours post-transfection cell-culture supernatant was collected and clarified by centrifugation at 13,000 rpm for 10 minutes and pellet discarded. To obtain cell extract, cells were lysed by incubating on ice with chilled cell extraction buffer (Invitrogen) supplemented with cOmplete, mini protease inhibitor cocktail (Roche) and 1mM phenylmethylsulfonyl fluoride (PMSF; Sigma) for 30 minutes with vortexing every 10 minutes. Cell extract was then clarified by centrifugation at 13,000 rpm for 10 minutes and pellet discarded. Proteins in supernatant and cell lysate were separated by SDS-PAGE (Tris-Acetate 4–15% gels, Invitrogen), blotted onto nitrocellulose membranes (Bio-Rad) and detected using primary antibodies against CL-L1 (Generon; CSB-PA896556LA01HU, 2μg/mL) and GAPDH (Generon; CSB-PA00025A0Rb, 2μg/mL) with HRP-conjugated secondary antibodies (Dako). Blots were developed with enhanced chemiluminescence (Pierce).

### ELISA

To obtain cell extract for ELISA, cells were lysed by incubating on ice with chilled ELISA cell extraction buffer (100mM Tris; pH7.4, 150mM NaCl, 1mM EGTA, 1mM EDTA, 1% Triton X-100 and 0.5% sodium deoxycholate) supplemented with cOmplete, mini protease inhibitor cocktail (Roche) and 1mM PMSF (Sigma) for 30 minutes with vortexing every 10 minutes. Cell extract was then clarified by centrifugation at 13,000 rpm for 10 minutes and pellet discarded.

### Inmunofluorescence and immunohistochemistry

For cell immunofluorescence ATDC5 cells were fixed with cold methanol -20°C, washed with PBS and blocked for 1 hour with 1% BSA. Cells were incubated overnight with the following antibodies and concentrations: CL-L1/100 (Novus Biologicals H00010584-M01), CL-K1 (Novus Biologicals H00010584-M01), Laminin (Abcam, ab11575). Cells were washed with PBS and incubated for 1 hour with Mouse or Rabbit Alexa Fluor 488 and 568 secondary antibodies (1/1000) (ThermoFisher). E18.5 mouse embryos were harvested and fixed in 4% paraformaldehyde overnight at 4°C, dehydrated and embedded in paraffin. 10μm sections were cut. Slides were rehydrated and blocked with 5% BSA with 10% of sheep serum. The samples were incubated with a rabbit in house made CL-L1 primary antibody (1/100) overnight at 4°C, washed in PBS and developed with a Horseradish peroxidase conjugated secondary antibody and diaminobenzidine staining.

### Web resources

1000 Genomes, http://www.1000genomes.org

Ensembl Genome Browser, http://www.ensembl.org/index.html

ExAC Browser, http://exac.broadinstitute.org/

OMIM, http://www.omim.org/

PolyPhen-2, http://genetics.bwh.harvard.edu/pph2/

SIFT, http://sift.bii.a-star.edu.sg/

### Ethics statement

All work involving human subject research was approved by the UCL-ICH/Great Ormond Street Hospital Research Ethics Committee (08/H0713/82) (REC reference 08/H0713/82, Protocol number HBD2008v1).All patients and families included in this work have given written consent for the use of their biological samples for research purposes under the HT act 2004 ethics committee. All animal work has been conducted under the UK Home Office regulation, Animals (Scientific Procedures) Act 1986 and was approved by the Home Office with Procedure Project License PPL number 70/7892.

## Supporting information

S1 Fig CL-L1 western blot after transfection of HEK293 cells with *COLEC10* cDNA. Different constructs of *colec10* containing wild-type (WT), c.25C>T; p.Arg9Ter c.25C>T, p.Gly77Glufs*66, c.528C>G and c.528C>G, p.Cys176Trp cDNAs were transfected. CL-L1 was found in wild-type and c.528C>G, p.Cys176Trp pellets, however only the supernatant of the wild-type construct contained CL-L1.
(TIF)

S1 Movie HeLa cells invading an agarose spot containing PBS for 48 hours. (AVI)

S2 Movie HeLa cells invading an agarose spot containing 0.015 μg of BSA for 48 hours. (AVI)

S3 Movie HeLa cells invading an agarose spot containing 0.015 μg of CL-L1 for 48 hours. (AVI)

S1 Table ELISA results for CL-L1 in cells pellets and supernatants after *COLEC10* mutations constructs were transfected. (PDF)

S1 Methods Table Table showing *MASP1/3*, *COLEC10* and *COLEC11* individual exons mean coverage and percentage of exons covered above 15x in Family 19. Coverage was sufficient to detect all variants in the coding regions of the genes.
(PDF)

S2 Methods Table Variant filtering criteria for family 19 exomes. (PDF)

S3 Methods Table Ingenuity filter pipeline used for family 19 exomes. (PDF)

S4 Methods Table Final list of genes containing variants identified at the end of the pipeline for family 19 exomes. (XLS)

S5 Methods Table Primers sequences used in the study. (PDF)

## References

[1] Titomanlio L, Bennaceur S, Bremond-Gignac D, Baumann C, Dupuy O, et al (2005) Michels syndrome, Carnevale syndrome, OSA syndrome, and Malpuech syndrome: variable expression of a single disorder (3MC syndrome)? Am J Med Genet A 137A: 332–335. 10.1002/ajmg.a.30878 16096999

[2] Leal GF, Silva EO, Duarte AR, Campos JF (2008) Blepharophimosis, blepharoptosis, defects of the anterior chamber of the eye, caudal appendage, radioulnar synostosis, hearing loss and umbilical anomalies in sibs: 3MC syndrome? Am J Med Genet A 146A: 1059–1062. 10.1002/ajmg.a.32252 18266249

[3] Mingarelli R, Castriota Scanderbeg A, Dallapiccola B (1996) Two sisters with a syndrome of ocular, skeletal, and abdominal abnormalities (OSA syndrome). J Med Genet 33: 884–886. 8933348 10.1136/jmg.33.10.884 PMC1050774

[4] Rooryck C, Diaz-Font A, Osborn DP, Chabchoub E, Hernandez-Hernandez V, et al (2011) Mutations in lectin complement pathway genes COLEC11 and MASP1 cause 3MC syndrome. Nat Genet 43: 197–203. 10.1038/ng.757 21258343 PMC3045628

[5] Atik T, Koparir A, Bademci G, Foster J 2nd, Altunoglu U, et al (2015) Novel MASP1 mutations are associated with an expanded phenotype in 3MC1 syndrome. Orphanet J Rare Dis 10: 128 10.1186/s13023-015-0345-3 26419238 PMC4589207

[6] Urquhart J, Roberts R, de Silva D, Shalev S, Chervinsky E, et al (2016) Exploring the genetic basis of 3MC syndrome: Findings in 12 further families. Am J Med Genet A 170: 1216–1224. 10.1002/ajmg.a.37564 26789649

[7] Gardner OK, Haynes K, Schweitzer D, Johns A, Magee WP, et al (2016) Familial Recurrence of 3MC Syndrome in Consanguineous Families: A Clinical and Molecular Diagnostic Approach With Review of the Literature. Cleft Palate Craniofac J. 10.1597/15-151 27356087

[8] Troldborg A, Thiel S, Jensen L, Hansen S, Laska MJ, et al (2015) Collectin liver 1 and collectin kidney 1 and other complement-associated pattern recognition molecules in systemic lupus erythematosus. Clin Exp Immunol 182: 132–138. 10.1111/cei.12678 26154564 PMC4608502

[9] Selman L, Hansen S (2012) Structure and function of collectin liver 1 (CL-L1) and collectin 11 (CL-11, CL-K1). Immunobiology 217: 851–863. 10.1016/j.imbio.2011.12.008 22475410

[10] Henriksen ML, Brandt J, Andrieu JP, Nielsen C, Jensen PH, et al (2013) Heteromeric complexes of native collectin kidney 1 and collectin liver 1 are found in the circulation with MASPs and activate the complement system. J Immunol 191: 6117–6127. 10.4049/jimmunol.1302121 24174618

[11] Ma YJ, Skjoedt MO, Garred P (2013) Collectin-11/MASP complex formation triggers activation of the lectin complement pathway—the fifth lectin pathway initiation complex. J Innate Immun 5: 242–250. 10.1159/000345356 23220946 PMC6741501

[12] Axelgaard E, Jensen L, Dyrlund TF, Nielsen HJ, Enghild JJ, et al (2013) Investigations on collectin liver 1. J Biol Chem 288: 23407–23420. 10.1074/jbc.M113.492603 23814060 PMC3743509

[13] Hansen S, Selman L, Palaniyar N, Ziegler K, Brandt J, et al (2010) Collectin 11 (CL-11, CL-K1) is a MASP-1/3-associated plasma collectin with microbial-binding activity. J Immunol 185: 6096–6104. 10.4049/jimmunol.1002185 20956340

[14] Degn SE, Jensen L, Olszowski T, Jensenius JC, Thiel S (2013) Co-complexes of MASP-1 and MASP-2 associated with the soluble pattern-recognition molecules drive lectin pathway activation in a manner inhibitable by MAp44. J Immunol 191: 1334–1345. 10.4049/jimmunol.1300780 23785123

[15] Venkatraman Girija U, Furze CM, Gingras AR, Yoshizaki T, Ohtani K, et al (2015) Molecular basis of sugar recognition by collectin-K1 and the effects of mutations associated with 3MC syndrome. BMC Biol 13: 27 10.1186/s12915-015-0136-2 25912189 PMC4431178

[16] Bayarri-Olmos R, Hansen S, Henriksen ML, Storm L, Thiel S, et al (2015) Genetic variation of COLEC10 and COLEC11 and association with serum levels of collectin liver 1 (CL-L1) and collectin kidney 1 (CL-K1). PLoS One 10: e0114883 10.1371/journal.pone.0114883 25710878 PMC4339841

[17] Fish JL (2015) Developmental mechanisms underlying variation in craniofacial disease and evolution. Dev Biol. 10.1016/j.ydbio.2015.12.019 26724698

[18] Kitazawa T, Fujisawa K, Narboux-Neme N, Arima Y, Kawamura Y, et al (2015) Distinct effects of Hoxa2 overexpression in cranial neural crest populations reveal that the mammalian hyomandibular-ceratohyal boundary maps within the styloid process. Dev Biol 402: 162–174. 10.1016/j.ydbio.2015.04.007 25889273

[19] Minoux M, Rijli FM (2010) Molecular mechanisms of cranial neural crest cell migration and patterning in craniofacial development. Development 137: 2605–2621. 10.1242/dev.040048 20663816

[20] Lan Y, Xu J, Jiang R (2015) Cellular and Molecular Mechanisms of Palatogenesis. Curr Top Dev Biol 115: 59–84. 10.1016/bs.ctdb.2015.07.002 26589921 PMC4663457

[21] Suzuki J, Osumi N (2015) Neural crest and placode contributions to olfactory development. Curr Top Dev Biol 111: 351–374. 10.1016/bs.ctdb.2014.11.010 25662265

[22] Maj E, Kunneke L, Loresch E, Grund A, Melchert J, et al (2016) Controlled levels of canonical Wnt signaling are required for neural crest migration. Dev Biol. 10.1016/j.ydbio.2016.06.022 27341758

[23] Xavier GM, Seppala M, Barrell W, Birjandi AA, Geoghegan F, et al (2016) Hedgehog receptor function during craniofacial development. Dev Biol 415: 198–215. 10.1016/j.ydbio.2016.02.009 26875496

[24] Carmona-Fontaine C, Theveneau E, Tzekou A, Tada M, Woods M, et al (2011) Complement fragment C3a controls mutual cell attraction during collective cell migration. Dev Cell 21: 1026–1037. 10.1016/j.devcel.2011.10.012 22118769 PMC3272547

[25] Leslie JD, Mayor R (2013) Complement in animal development: unexpected roles of a highly conserved pathway. Semin Immunol 25: 39–46. 10.1016/j.smim.2013.04.005 23665279 PMC3989114

[26] Broders-Bondon F, Paul-Gilloteaux P, Gazquez E, Heysch J, Piel M, et al (2016) Control of the collective migration of enteric neural crest cells by the Complement anaphylatoxin C3a and N-cadherin. Dev Biol 414: 85–99. 10.1016/j.ydbio.2016.03.022 27041467 PMC4937886

[27] Cho MS, Rupaimoole R, Choi HJ, Noh K, Chen J, et al (2016) Complement Component 3 Is Regulated by TWIST1 and Mediates Epithelial-Mesenchymal Transition. J Immunol 196: 1412–1418. 10.4049/jimmunol.1501886 26718342 PMC4724537

[28] Szabo A, Cobo I, Omara S, McLachlan S, Keller R, et al (2016) The Molecular Basis of Radial Intercalation during Tissue Spreading in Early Development. Dev Cell 37: 213–225. 10.1016/j.devcel.2016.04.008 27165554 PMC4865533

