# Supplementary figures and images for: COLEC10 is mutated in 3MC patients and regulates early craniofacial development
Source: PLoS Genet. 2017 Mar 16;13(3):e1006679. doi: 10.1371/journal.pgen.1006679 (PMC5373641; doi:10.1371/journal.pgen.1006679)

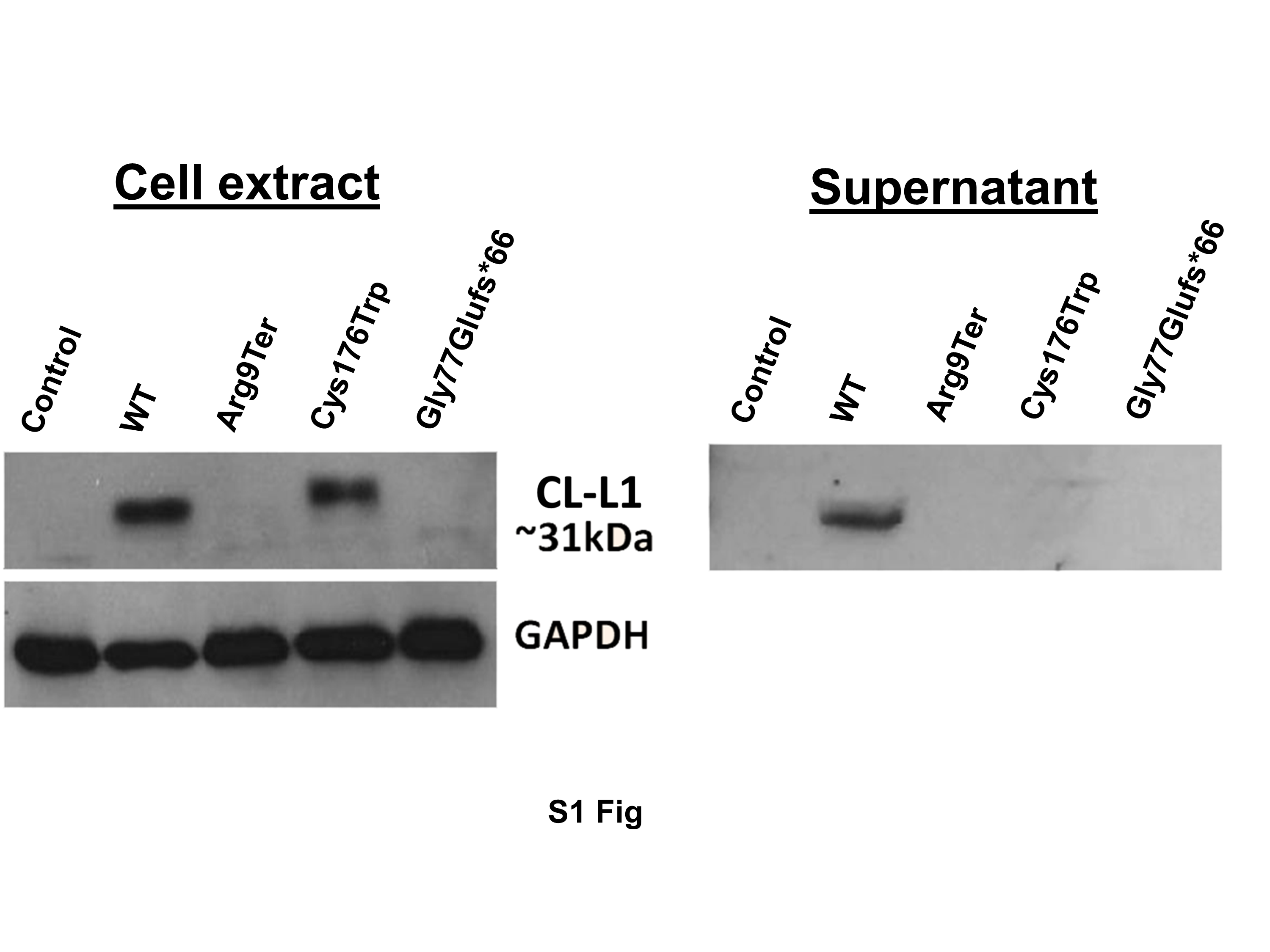

Supplement: S1 Fig — Different constructs of colec10 containing wild-type (WT), c.25C>T; p.Arg9Ter c.25C>T, p.Gly77Glufs*66, c.528C>G and c.528C>G, p.Cys176Trp cDNAs were transfected. CL-L1 was found in wild-type and c.528C>G, p.Cys176Trp pellets, however only the supernatant of the wild-type construct contained CL-L1. (TIF) [file pgen.1006679.s001.tif]
