# Supplementary material for: COLEC10 is mutated in 3MC patients and regulates early craniofacial development
Source: PLoS Genet. 2017 Mar 16;13(3):e1006679. doi: 10.1371/journal.pgen.1006679 (PMC5373641; doi:10.1371/journal.pgen.1006679)
Supplement: S1 Table — (PDF) [file pgen.1006679.s005.pdf]

	Mean CL-L1 concentration ± S.E.M. (ng/mL)			
	**HeLa**		**HEK293**	
	Cell extract	Supernatant	Cell extract	Supernatant
Untransfected	22.9±0.4	<1.0	23.4±0.3	<1.0
COLEC10 (WT)	1823.5±7.4	200.3±1.5	632.0±3.6	390.2±4.1
COLEC10 (p.Arg9Ter)	29.2±0.2	<1.0	25.2±0.3	<1.0
COLEC10 (p.Cys176Trp)	2518.4±21.2	12.5±0.2	1302.7±3.5	5.7±0.1
COLEC10 (p.Gly77Glufs*66)	13.8±0.1	<1.0	31.7±0.4	<1.0
