# Supplementary material for: COLEC10 is mutated in 3MC patients and regulates early craniofacial development
Source: PLoS Genet. 2017 Mar 16;13(3):e1006679. doi: 10.1371/journal.pgen.1006679 (PMC5373641; doi:10.1371/journal.pgen.1006679)
Supplement: S1 Methods Table — Coverage was sufficient to detect all variants in the coding regions of the genes. (PDF) [file pgen.1006679.s006.pdf]

Target	Exon	Gene	Proband 19.1_average coverage	Proband 19.1_%_above_15x	Dad 19.3_average coverage	Dad 19.3_%_above_15x	Mum_ 19.4_average coverage	Mum 19.4_%_above_15x
3:186964142-186965173	MASP1_5'UTR	MASP1	3.32	6.6	4.35	16.6	4.09	13.9
3:186968039-186968117	MASP1_exon1	MASP1	43.09	100	51.59	100	30.57	100
3:186969422-186969540	MASP1_exon2	MASP1	25.65	100	22.5	98.3	16.26	59.7
3:186970956-186971103	MASP1_exon3	MASP1	68.13	100	64.8	100	61.72	100
3:186974452-186974648	MASP1_exon4	MASP1	65.21	100	61.77	100	76.94	100
3:186978529-186978660	MASP1_exon5	MASP1	23.33	90.2	25.13	100	29.36	97
3:186980331-186980508	MASP1_exon6	MASP1	18.71	93.3	25.44	92.7	19.42	85.4
3:187003613-187003844	MASP1_exon7	MASP1	131.52	100	163.15	100	140.79	100
3:187009416-187009810	MASP1_3'UTR	MASP1	129.97	86.3	158.49	87.3	152.72	90.9
8:120079424-120079668	COLEC10_5'UTR	COLEC10	75.29	100	84.46	100	80.26	100
8:120101919-120101990	COLEC10_exon1	COLEC10	25.31	100	46.72	100	51.24	100
8:120103388-120103459	COLEC10_exon2	COLEC10	85.56	100	116.54	100	112.86	100
8:120114587-120114640	COLEC10_exon3	COLEC10	60.83	100	67.26	100	51.22	100
8:120116039-120116134	COLEC10_exon4	COLEC10	202.04	100	197.86	100	204.89	100
8:120118039-120119202	COLEC10_3'UTR	COLEC10	30.61	45.4	46.64	49.3	37.52	45.9
2:3642637-3642758	COLEC11_5'UTR	COLEC11	0.27	0	0	0	0.66	0
2:3651905-3652060	COLEC11_exon1	COLEC11	115.54	100	141.91	100	112.01	100
2:3660901-3660972	COLEC11_exon2	COLEC11	23.58	100	36.13	100	24.17	100
2:3673604-3673682	COLEC11_exon3	COLEC11	78.59	100	84.94	100	60.65	100
2:3685123-3685194	COLEC11_exon4	COLEC11	77.4	100	78.56	100	87.43	100
2:3687868-3687921	COLEC11_exon5	COLEC11	23.85	100	30.44	100	13.39	24.1
2:3691034-3691129	COLEC11_exon6	COLEC11	49.58	100	65.68	100	46.58	100
2:3691317-3692047	COLEC11_3'UTR	COLEC11	34.02	70.7	41.03	70.9	35.65	73.2
Genome Build GRCh37/hg19								

S1 Methods Table
