# Supplementary material for: COLEC10 is mutated in 3MC patients and regulates early craniofacial development
Source: PLoS Genet. 2017 Mar 16;13(3):e1006679. doi: 10.1371/journal.pgen.1006679 (PMC5373641; doi:10.1371/journal.pgen.1006679)
Supplement: S2 Methods Table — (PDF) [file pgen.1006679.s007.pdf]

S2 Methods Table. Variant filtering criteria for family 19 exomes

Analysis: **3MC**

Sample Table:
BarCode
UCLG367_new.vcf
UCLG368_new.vcf
UCLG369_new.vcf

**Name**	**Description**	**Subject**	**Status**
19.1	Affected	UCLG367	CASE
19.3	Dad	UCLG368	CONTROL
19.4	Mum	UCLG369	CONTROL

excluded that are observed with an allele frequency greater than or equal to 0.01% of the genomes in the 1000 genomes project OR greater than or equal to 0.01% of the NHLBI ESP exomes (All) OR greater than or equal to 0.5% of the AFC Frequency OR greater than or equal to 0.01% of the ExAC Frequency

kept that are experimentally observed to be associated with a phenotype: Pathogenic, Possibly Pathogenic OR Frameshift, in-frame indel, or stop codon change OR Missense OR disrupt splice site up to 2.0 bases into intron

kept with call quality at least 20.0 in cases or at least 20.0 in controls

kept which are homozygous OR compound_heterozygous AND occur in at least 1 of the case samples at the variant level in the Case samples AND not which are homozygous OR haploinsufficient OR hemizygous OR compound_heterozygous AND occur in at least 1 of the control samples at the variant level in the Control Samples

kept that are within 2 hops upstream and that are known or predicted to affect: 3MC syndrome type 1, 3MC syndrome type 2, 3MC1, 3MC2 or diseases consistent with these phenotypes

Ingenuity Variant Analysis version 4.2.20170105

Content versions: CADD (v1.3), SIFT (2016-02-22), EVS (ESP6500SI-V2), Allele Frequency Community (2016-08-26), JASPAR (2013-11), Ingenuity Knowledge Base (Krikkit 170203.002), Vista Enhancer (2012-07), Clinical Trials (Krikkit 170203.002), BSIFT (2016-02-22), TCGA (2013-09-05), PolyPhen-2 (v2.2.2), 1000 Genome Frequency (phase3v5b), Clinvar (2016-09-01), COSMIC (v78), ExAC (0.3.1), HGMD (2016.3), PhyloP (2009-11), DbSNP (147), TargetScan (6.2)
