# Supplementary material for: COLEC10 is mutated in 3MC patients and regulates early craniofacial development
Source: PLoS Genet. 2017 Mar 16;13(3):e1006679. doi: 10.1371/journal.pgen.1006679 (PMC5373641; doi:10.1371/journal.pgen.1006679)
Supplement: S3 Methods Table — (PDF) [file pgen.1006679.s008.pdf]

S3 Methods Table. Ingenuity filter pipeline

Filter	Feature (For Recessive Analysis)	TH
Confidence	Call quality (Phred Score – Base call accuracy>99%)	20.00
Common Variants	1000 Genomes Project	0.01
	Complete Genomics genomes	0.50
	NHLBI ESP exomes	0.01
	EXAC	0.01
Predicted Deleterious	Experimentally pathogenic	✓
	Likely pathogenic	✓
	Uncertain significance	✓
	**Associated with gain of function**	
	Established in Literature	
	Gene fusion	
	Inferred activating mutation (Ingenuity)	
	Predicted gain of function by BSIFT	
	**Associated with loss of function**	
	Frameshift, in-frame indel, stop codon change	✓
	Missense	✓
	Likely splice site loss up to 2 bases into intron	✓
Genetic Analysis	**Keep variants only if in at least one case sample:**	
	Homozygous	✓
	Compound Heterozygous	✓
	Haploinsufficient	
	Hemizygous	
	**Exclude variants if in 2 or more of the control samples**	
	Filter out haploinsufficient and hemizygous variant	✓
	Filter out homozygous and compound heterozygous	✓
	Exclude variants in MUC and HLA loci	✓
	Present in dbSNP database	
Biological Context	3MC syndrome type 1 (*MASP1*)	✓
	3MC syndrome type 2 (*COLEC11*)	✓
