# Supplementary material for: COLEC10 is mutated in 3MC patients and regulates early craniofacial development
Source: PLoS Genet. 2017 Mar 16;13(3):e1006679. doi: 10.1371/journal.pgen.1006679 (PMC5373641; doi:10.1371/journal.pgen.1006679)
Supplement: S5 Methods Table — (PDF) [file pgen.1006679.s010.pdf]

S5 Methods Table. Primer sequences

Primer name	Sequence (5'-3')
hCOLEC10Arg9Ter Forward	GAGGATAAATTGGTTTCTTCAAAGCAAGGATGCAAAGCCAT
hCOLEC10Arg9Ter Reverse	ATGGCTTTGCATCCTTGCTTTGAAGAAACCAATTTATCCTC
hCOLEC10Gly77Glufs*66 Forward	CATGGGGCCGAAAGGAATTAAGGAGAACTGGG
hCOLEC10Gly77Glufs*66 Reverse	CCCAGTTCTCCTTAATTCCTTTCGGCCCCATG
hCOLEC10Cys176Trp Forward	CACCCCGAATCCTCCAGTGGGTTAGGG
hCOLEC10Cys176Trp Reverse	CCCTAACCCACTGGAGGATTCGGGGTG
